# Efficacy and Selectivity of FGF2-Saporin Cytosolically Delivered by PCI in Cells Overexpressing FGFR1

**DOI:** 10.3390/cells10061476

**Published:** 2021-06-12

**Authors:** Aurora K. Vikan, Michal Kostas, Ellen Margrethe Haugsten, Pål K. Selbo, Jørgen Wesche

**Affiliations:** 1 Department of Tumor Biology, Institute for Cancer Research, The Norwegian Radium Hospital, Oslo University Hospital, Montebello, 0379 Oslo, Norway; auroravikan@hotmail.com (A.K.V.); Michal.Janusz.Kostas@rr-research.no (M.K.); Ellen.M.Haugsten@rr-research.no (E.M.H.); 2 Centre for Cancer Cell Reprogramming, Institute of Clinical Medicine, Faculty of Medicine, University of Oslo, Montebello, 0379 Oslo, Norway; 3 Department of Radiation Biology, Institute for Cancer Research, The Norwegian Radium Hospital, Oslo University Hospital, Montebello, 0379 Oslo, Norway

**Keywords:** FGFR1, PCI, PDT, HSPG, FGF-SAP

## Abstract

Fibroblast growth factor receptors (FGFRs) have become an attractive target in cancer research and therapy due to their implication in several cancers. Limitations of current treatment options require a need for additional, more specific and potent strategies to overcome cancers driven by FGFRs. Photochemical internalization (PCI) is a light-controlled method for cytosolic delivery of drugs that are entrapped in endosomes and lysosomes. We here evaluated the efficacy and selectivity of PCI of FGF2-saporin (FGF-SAP) in cells overexpressing FGFR1. FGF-SAP is a conjugate of FGF2 and the highly cytotoxic ribosome-inactivating protein (RIP) saporin, which is used as payload to eliminate cancer cells. Evaluation of the targeting effect of PCI of FGF-SAP was done by comparing the cytotoxic response in osteosarcoma cells with very low levels of FGFR1 (U2OS) to cells overexpressing FGFR1 (U2OS-R1). We demonstrate that PCI greatly enhances cytotoxicity of the drug showing efficient cell killing at pM concentrations of the drug in U2OS-R1 cells. However, U2OS cells were also sensitive to the toxin after PCI. Binding experiments using confocal microscopy and Western blotting techniques indicate that FGF-SAP is taken up by cells through heparan sulfate proteoglycans (HSPGs) in U2OS cells. We further show that the cytotoxicity of FGF-SAP in U2OS cells was reduced when cells were co-treated with heparin to compete out binding to HSPG, demonstrating that the cytotoxic effect was due to internalization by HSPGs. We conclude that to prevent off-target effects of FGF-based toxins, it will be necessary to circumvent binding to HSPGs, for example by mutating the binding site of FGF2 to HSPGs.

## 1. Introduction

Over a century ago Paul Ehrlich introduced the concept of “the magic bullet”. The idea was to develop a drug that would precisely target disease-causing agents [1]. Since then, much effort has been done to develop drugs that specifically target cancer cells. The main categories for targeted therapies are currently small molecule inhibitors (e.g., gefitinib inhibiting epidermal growth factor receptor (EGFR) signaling) and monoclonal antibodies (mAbs) (e.g., trastuzumab against the human epidermal growth factor receptor 2 (HER2) receptor). However, treatment is often impaired by nonspecific drug distribution, lack of specificity to the target site, and acquired resistance [2,3].

The major objective of targeted cancer therapy is reducing side effects and enhancing drug accumulation at the target site for effective and selective cancer eradication [3]. Several strategies can be employed for targeted drug delivery. One such strategy is to exploit the expression of specific receptors on the surface of cancer cells. Discrimination between healthy and cancerous tissue is possible by applying a targeting ligand that binds efficiently to these surface markers [3]. By coupling a ligand to a toxic moiety, receptor–ligand interaction triggering endocytosis can deliver a drug to the target site [3,4]. Various ligands can be used, such as hormones, growth factors, or antibodies. The most common drug carriers are, however, mAbs. Among the very few antibody–drug conjugates (ADCs) that have been approved by the U.S. Food and Drug Administration (FDA) are, for example, brentuximab vedotin (Adcetris™) for treatment of relapsed Hodkin’s lymphoma and systemic anaplastic large cell lymphoma [5], and ado-trastuzumab ematisine (Kadcyla™) for HER2-positive metastatic breast cancer [6].

The FGFR family consists of four signaling RTKs, termed FGFR1-FGFR4 [7,8]. They are structurally similar to other RTKs [9]. The extracellular domain consists of two or three immunoglobulin-like domains (D1–D3). Upon dimerization caused by the ligands (FGFs), the two receptors will interact directly through D2, which also contains a heparin binding site [10,11]. D2–D3 constitutes the FGF binding site. In addition to the extracellular domain, FGFRs contain a single transmembrane helix part and an intracellular split, catalytically active kinase domain [8,12]. FGFR dimerization leads to activation of the tyrosine kinase in the intracellular part and initiation of several downstream signaling pathways [7,12].

FGFs also have affinity for the closely related heparin and cell surface heparan sulfate proteoglycans (HSPGs) [12,13]. Both heparin and HS are common glycosaminoglycans in proteoglycans, and regulate several biological processes by reacting with a number of molecules [14]. HSPGs play an important regulatory role in FGF-signaling. FGFs with high affinity to HSPGs are readily sequestered, thereby limiting their diffusion through the extracellular matrix. Thus, HSPGs act as storage and protect FGFs from degradation. Moreover, HSPGs facilitate the activation of and signaling through FGFRs by stabilizing ligand-receptor binding [10].

Aberrant activation of FGFRs is observed in a number of cancers [7,8]. Alterations in FGFRs include activating mutations, fusion proteins and receptor overexpression. Constitutively active receptors may occur by activating mutations in the kinase domain, mutations leading to ligand-independent dimerization or even chromosomal rearrangement resulting in dimerized FGFRs and ligand-independent activation. Furthermore, gene amplification or abnormal transcriptional regulation may lead to upregulation of FGFRs, resulting in increased sensitivity to FGFs and subsequently increased FGFR signaling [15]. The majority of FGFR alterations are receptor overexpression, followed by mutations and rearrangements [16]. 

Large efforts have been devoted to developing strategies to target FGFRs and their signaling pathways [12]. Tyrosine kinase inhibitors are the most common approach in targeting FGFR aberrations. Other efforts include mAbs, ADCs, aptamers and ligand traps [12,17]. However, the efficacies of anti-FGFR therapies have been variable. Clinical trials have revealed a complexity in targeting FGFRs, challenged by factors such as resistance to therapy, secondary FGFR alterations, bypass of signaling pathways and intratumoral heterogeneity [18,19]. Still, FGFR aberrations are observed in many cancers and remain an important target.

PCI was developed and first reported by Berg et al. in 1999. It was proposed as a new technology for site-specific delivery of macromolecules into the cytosol [20]. The major objective of selective and targeted cancer therapy is reducing side effects as well as enhancing drug accumulation at the target site. Macromolecules that exert their effect intracellularly are of great therapeutic interest but are often limited by ineffective internalization as they are transported to and degraded in lysosomes. In order to reach their intracellular targets, the molecules must escape from endosomal and/or lysosomal compartments [20,21]. PCI is based on a photosensitizer that, due to its amphiphilic nature, accumulates in endo- and lysosomal membranes. Photochemical treatment induces ROS-generation resulting in disintegration of these membranes, which is the basis of photodynamic therapy (PDT). In PCI, the subsequent release of the endosomal content into the cytosol of light exposed cells is exploited to deliver drugs intracellularly.

Ideal candidates for PCI include therapeutic agents that are normally ineffectively delivered to the cytosol. One such example is type I ribosome inactivating proteins (RIPs) [20]. RIPs from plants are enzymes that exert N-glycosidase activity. By depurinating a specific nucleotide in the large subunit of ribosomes, protein translation is inhibited. This will in turn lead to cell death. There are two types of RIPs. Type II RIPs consist of an A and B chain, e.g., ricin, which is one of the most potent natural toxins. The A chain hold the enzymatic activity, while the B chain enables translocation into the cell. On the other hand, type I RIPs lack the B chain and does not possess a way of entering the cell [22].

The lack of the translocation domain in type I RIPs (e.g., saporin and gelonin) make them far less toxic. When access is gained to the cytosol, however, they have similar RIP activity. Type I RIPs are mainly internalized by pinocytosis, which is both non-selective and inefficient [22]. However, their potential can be exploited by linking the A chain to a targeting vessel, such as antibodies or targeting ligands. Previous in vitro and in vivo studies of PCI of saporin and gelonin-based immunotoxins and ligand toxins targeting EGFR [23,24], vascular endothelial growth factor receptor (VEGFR) [25], CSPG4 [26,27], CD133 [28,29] and HER2 [30] have been shown to enhance the delivery and efficacy of these cytotoxic agents.

The aim of the present study was to evaluate PCI of FGF2-saporin (FGF-SAP), a conjugate of FGF2 and saporin [31], as a strategy to target and achieve cytotoxic effects in cancer cells overexpressing FGFRs. FGF-SAP has been used previously to eliminate fibroblasts in primary cultures [32] and has shown effects on cancer cells expressing FGFRs both in vitro and in vivo [33]. We wanted here to investigate whether PCI could potentiate the cytotoxic effect of FGF-SAP.

We show that PCI can successfully deliver FGF-SAP into cells in order for saporin to exert its toxic effect at pM concentrations. However, we also find that HSPGs-mediated uptake of FGF-SAP causes a challenge with respect to the selectivity of FGF-SAP against FGFR overexpressing cells. We conclude that for FGFR-specific cell killing in vitro*,* co-incubation with heparin can be used to avoid binding to HSPGs, while in vivo*,* mutation of the heparin-binding site in FGF2 could be a possible way to avoid internalization by HSPGs.

## 2. Materials and Methods

### 2.1. Cells

The human osteosarcoma cell lines U2OS (ATCC, Manassas, VA, USA) and U2OS-R1 (established by Haugsten et al. [34] were maintained in Dulbecco’s Modified Eagle Medium GlutaMAX™-I (Thermo Fisher Scientific, Waltham, MA, USA) supplemented with 10% fetal bovine serum (FBS, Thermo Fisher Scientific), 100 U/mL penicillin (Sigma-Aldrich, St. Louis, MO, USA) and 100 μg/mL streptomycin (Sigma-Aldrich) and incubated at 37 °C in a humidified atmosphere containing 5% CO_2_. Cells were routinely assessed for mycoplasma infections.

### 2.2. Materials, Antibodies, and Compounds

FGF-SAP (Advanced Targeting Systems, Carlsbad, CA, USA) is a chemical conjugate of FGF2 and saporin. 

Unconjugated saporin, SAP (Advanced Targeting Systems, Carlsbad, CA, USA), served as control for the targeted ligand toxin. There are approximately 1.5 molecules of saporin conjugated per molecule of FGF2, which was accounted for when treating the cells with saporin versus FGF-SAP.

The plasmid pDEST15-GST-FGF2 was a generous gift from Dr. Malgorzata Zakrzewska (Department of Protein Engineering, Faculty of Biotechnology, University of Wroclaw, Poland) and recombinant GST-FGF2 was prepared as previously described [35].

The following antibodies were used: mouse anti-γ-tubulin (T6557) from Sigma-Aldrich (St. Louis, MO, USA), mouse anti-GST (Sc-138) and rabbit anti-GST (Sc-459) from Santa Cruz Biotechnology (Dallas, TX, USA), mouse anti-Lamp1 (1D4B) from Developmental Studies Hybridoma Bank. HRP-conjugated secondary antibodies were from Dako (Glostrup, Denmark). Fluorescently labeled secondary antibodies were from Jackson ImmunoResearch Laboratories (Cambridgeshire, UK).

Hoechst 33,258 and heparin were from Sigma Aldrich (St. Louis, MO, USA). cOmplete EDTA-free protease inhibitor cocktail was from Roche (Basel, Switzerland).

### 2.3. PDT and PCI Treatment

PDT and PCI were performed using the photosensitizer fimaporfin (TPCS_2a_), (PCI Biotech, Oslo, Norway). TPCS_2a_ absorbs light most efficiently in the blue area (λ_max_ ≈ 420 nm), but also has an in vivo/clinically relevant peak in the red area (λ_max_ ≈ 650 nm) [36]. Cells were illuminated using the light lamp LumiSource™ (PCI Biotech, which consists of four light tubes (4 × 18 W Osram L 18/67, Blue) emitting blue light with a peak wavelength of approximately 435 nm at a fluence rate of 11.5 mW/cm^2^ (1 min light exposure = 0.69 J/cm^2^). Except during illumination, all work with TPCS_2a_ was performed in subdued light.

A total of 3000 cells/well were seeded in 96-well plates (Nunclon™ Delta Surface, Thermo Fisher Scientific) and placed for attachment overnight. The cells were then incubated with TPCS_2a_ for 18 h overnight, prior to 2× wash and a 4-hour chase period to remove the photosensitizer from the plasma membrane before illumination. PCI included co-incubation of FGF-SAP with the photosensitizer overnight, or in the photosensitizer-free medium 4 h prior to light exposure. Cell viability was assessed approximately 48 h after illumination.

PDT and PCI data are presented as means of three independent experiments plus/minus standard error of the mean (SEM). The data were normalized to control (untreated cells)

### 2.4. Viability Assay

The CellTiter 96^®^ AQueous One Solution Cell Proliferation Assay (Promega Corporation, Madison, WI, USA) was used to assess the cytotoxic responses to treatments according to the manufactuer’s protocol. The absorbance at 490 nm was recorded by PowerWave™ XS2 Microplate Spectrophotometer (BioTek Instruments, Inc., Winooski, VT, USA) and analyzed with Gen5™ Data Analysis Software (BioTek Instruments, Inc.).

### 2.5. Flow Cytometry

A total of 100,000 cells/well were seeded in 6-well plates (Nunclon™ Delta Surface, Thermo Fisher Scientific) and left for attachment. After approximately 24 h, the medium was exchanged with fresh medium containing 0.2 μg/mL TPCS_2a_. Cells were incubated with photosensitizer for 22 h or alternatively, cells were kept for 18 h before washing and further incubation for a 4-hour chase period in drug-free medium. Next, cells were detached by trypsination followed by a 5-min centrifugation at 235× *g* at room temperature (RT). The cells were then washed with PBS before a second centrifugation. The supernatant was removed, and the pellet resuspended in standard culture medium. The cell suspension was filtrated and collected in designated flow tubes. Treated cells were protected from light until running the samples.

Flow cytometry was performed with BD™ LSR II flow cytometer (BD Biosciences, Franklin Lakes, NJ, USA). Violet laser (407 nm) was used to excite TPCS2a with the following filter settings for detection of emitted fluorescence: bandpass filter 660/20 nm (650–670 nm) and longpass filter 635 nm. Dead and live cells were determined by SSC/FSC. Data were analyzed using the software program FlowJo™ (Three Stars Inc., Ashland, VA, USA).

### 2.6. Immunofluorescence Confocal Microscopy

A total of 200,000 cells/well were seeded onto 10 mm round coverslips in 6-well plates and left for attachment overnight. The following day, the cells were treated with culture medium containing 100 ng/mL GST-FGF2, with or without 20 U/mL heparin (Sigma-Aldrich), for 30 min or 4 h at 37 °C in a humidified atmosphere containing 5% CO_2_. Treatment was ended by a 3× wash in PBS. Cells were fixed with 10% Neutral Buffered Formalin solution (Sigma-Aldrich) for 10 min at RT. The fixative was removed by washing 3× with PBS.

Cells were permeabilized using 0.05% saponin (Sigma-Aldrich) in PBS and left for 10 min at RT before staining with primary antibody and a secondary antibody coupled to a fluorophore.

The coverslips were immersed in Hoechst solution for 10–15 s followed by immersion in ultrapure deionized water (Milli-Q H2O) and mounted in ProLong™ Diamond Antifade Mountant (Invitrogen, Carlsbad, CA, USA). 

Images were acquired with a 63× objective on Zeiss LSM 710 confocal microscope (Carl Zeiss AG, Oberkochen, Germany). Processing and analysis of images was performed in ImageJ (version 1.52o) [37].

### 2.7. Western Blotting

A total of 100,000 cells/well were seeded in 12-well plates and left overnight for attachment. The cells were then treated with 600 ng/mL GST-FGF2 in the absence or presence of 50 U/mL heparin for 1 h at 4 °C. Next, the cells were washed 10× with ice cold PBS to remove unspecifically bound GST-FGF2. 

Subsequently, cells were lysed in lysis buffer (10 mM phosphate-Na pH 7.4, 100 mM NaCl, 1 mM Ethylenediaminetetraacetic acid (EDTA), 1% Triton X-100, protease inhibitors) for 5–10 min on ice. Lysates were then mixed with sample buffer, containing lithium dodecyl sulfate (LDS) at a pH of 8.4 (NuPAGE™ LDS Sample Buffer, Invitrogen) and reducing agent, containing 500 mM dithiothreitol (DTT) (NuPAGE™ Sample Reducing Agent, Invitrogen), and boiled at 95 °C for 5 min. 

Separation by gel electrophoresis was carried out using NuPAGE™ 4–12% Bis-Tris Protein Gels (Invitrogen) and NuPAGE™ MES SDS Running Buffer (50 mM MES, 50 mM Tris Base, 0.1% SDS, 1 mM EDTA, pH 7.3) (Invitrogen) at 150 V. Precision Plus Protein™ Dual Color Standards (Bio-Rad, Hercules, CA, USA) were included. Transfer was performed using the iBlot^®^ dry Blotting System (Invitrogen) using 20 V for 10 min.

The membrane was then incubated with primary antibodies followed by secondary antibodies conjugated to Horseradish Peroxidase (HRP). 

SuperSignal™ West Dura system (Thermo Fisher Scientific) was used to produce chemiluminescence that was captured using the imager system ChemiDoc™ (Bio-Rad). Images were analyzed and processed in Image Lab™ version 6.0.0 (Bio-Rad).

## 3. Results

### 3.1. Optimization of PCI Parameters

To investigate the specificity of PCI of FGF-SAP towards cells overexpressing FGFRs, we chose to use a cellular model system consisting of cells stably expressing FGFR1 (U2OS-R1) and compare the efficiency in these cells to the parental cell line (U2OS) with very low (non-detectable levels) of all FGFRs (FGFR1-4) [38]. This allowed us to compare the efficiency of FGF-SAP PCI in similar cellular settings with and without FGFR1.

**Figure 1:**
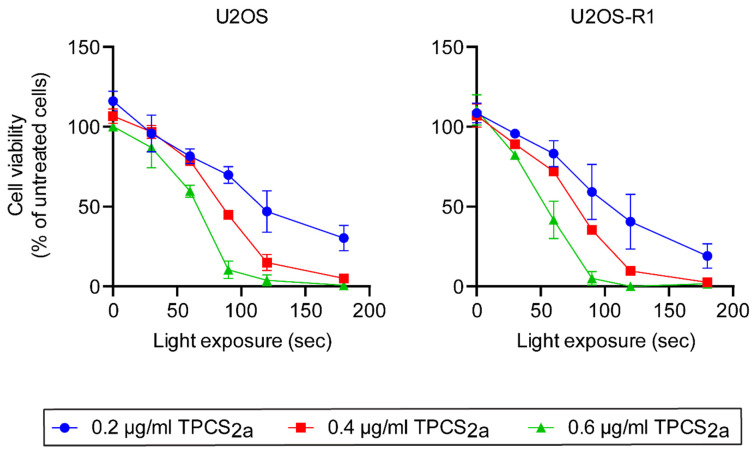
PDT of TPCS2a in U2OS and U2OS-R1 cells. Cells were incubated with 0.2, 0.4 or 0.6 µg/mL TPCS_2a_ for 18 h prior to a 4 h chase in photosensitizer-free medium and exposure to blue light for 0–180 s. Cell viability was assessed with MTS assay approximately 48 h after illumination. The data are presented as means relative to the mean of untreated cells (no TPCS_2a_ treatment). The graph represents the mean of three independent experiments with three replicate samples for each condition in each experiment. 30 s of blue light with 0.2 and 0.4 µg/mL TPCS2a in U2OS cells was only repeated twice (*n* = 2). Error bars ± SEM, *n* = 3.

First, the optimal blue light dose and concentration of the photosensitizer, TPCS_2a_, in U2OS and U2OS-R1 cells was investigated. To establish the optimal conditions, cytotoxic response of TPCS_2a_-PDT (TPCS_2a_ and blue light exposure in the absence of drug) was investigated (Figure 1). Cells were treated with different concentrations of TPCS_2a_ and illuminated for different periods of time. 

While the viability response curves for 0.4 and 0.6 μg/mL TPCS_2a_ decreased rapidly at longer illumination times, the viability of cells treated with 0.2 μg/mL decreased less at longer illumination times. Subjecting the cells to 0.2 μg/mL TPCS_2a_ and 120 s illumination decreased the cell viability to approximately 40–50% in both cell lines. Light doses causing 30–50% reduction in cell viability have previously been reported to be optimal for drug delivery by PCI. At these light doses, lysosomes are efficiently ruptured and release their content into the cytosol, allowing saporin to exert its toxic effect [39]. 0.2 μg/mL TPCS_2a_ and 120 s of light exposure were therefore selected for subsequent PCI experiments.

### 3.2. Cellular Uptake of TPCS_2a_

**Figure 2:**
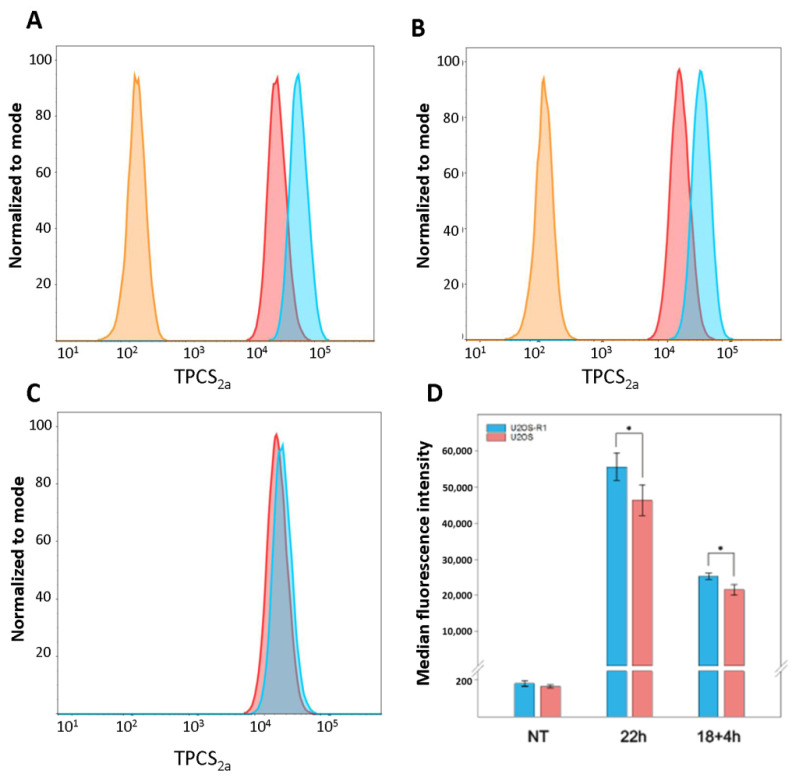
Cellular uptake of TPCS2a evaluated by flow cytometry. (**A**–**C**) Representative histograms from one experiment, (**D**) median fluorescence intensity (MFI) as an average from three independent experiments. Cells were incubated with 0.2 μg/mL TPCS2a for 22 h or 18 + 4 h (2× wash after 18 h). (**A**,**B**) U2OS-R1 and U2OS cells, respectively. Orange histograms represent untreated cells. Treated cells are shown in red (18 + 4 h) and blue (22 h). (**C**) U2OS (red) and U2OS-R1 (blue) when treated for 18 + 4 h from (**A**,**B**). (**D**) Average MFI determined on the basis of three independent experiments. Error bars ± S.D. * *p* < 0.05 (two-tailed Student’s *t*-test). Blue bars represent U2OS-R1 cells, red bars represent U2OS cells.

The PDT results in Figure 1 indicated that U2OS-R1 cells were slightly more sensitive to PDT as higher light doses (10–15%) were needed in order to obtain the same cytotoxic effect in U2OS cells as in U2OS-R1. The cellular uptake of TPCS_2a_ in U2OS-R1 and U2OS cells was therefore evaluated by flow cytometry (Figure 2). Cells were treated with 0.2 μg/mL TPCS_2a_ for 22 h. Alternatively, cells were treated with 0.2 µg/mL TPCS_2a_ for 18 h before the cells were subjected to a washing step and incubated further for 4 h. Total incubation time in both cases was 22 h.

The results reveal a large shift in median fluorescence intensity (MFI) compared to non-treated cells (Figure 2A,B). Moreover, we observed that more TPCS_2a_ was taken up into cells when cells were kept for 22 h than when they were kept for 18 h, and washed. Employing a 2× wash after 18 h of incubation decreased the median fluorescence intensity by approximately 50% in both U2OS-R1 and U2OS. More importantly, upon TPCS_2a_ treatment for 22 h, as well as upon TPCS_2a_ treatment for 18 h followed by washing and 4 more hours, U2OS cells internalized significantly less TPCS_2a_ than U2OS-R1 cells. After continuous incubation with TPCS_2a_ for 22 h, a small, but significant (*p* = 0.048), difference in fluorescence intensity between the cell lines was observed. When employing a 2× wash followed by a 4-hour chase period, thereby mimicking the PCI protocol described below, there was also a significant (*p* = 0.018) difference between the cell lines. This demonstrates that, indeed, U2OS-R1 cells have a slightly higher uptake of TPCS_2a_ relative to U2OS, which corresponds well with the difference in PDT-sensitivity. 

### 3.3. PCI of FGF-SAP

After having established the optimal concentration of photosensitizer and exposure of light, we proceeded to test whether PCI could increase cytosolic delivery and toxicity of FGF-SAP. The efficiency of PCI of FGF-SAP in U2OS and U2OS-R1 cells was compared to the efficiency of non-targeted saporin (SAP) and measured as cytotoxicity using the MTS assay.

#### 3.3.1. Cytotoxic Effects of PCI of SAP and FGF-SAP

**Figure 3:**
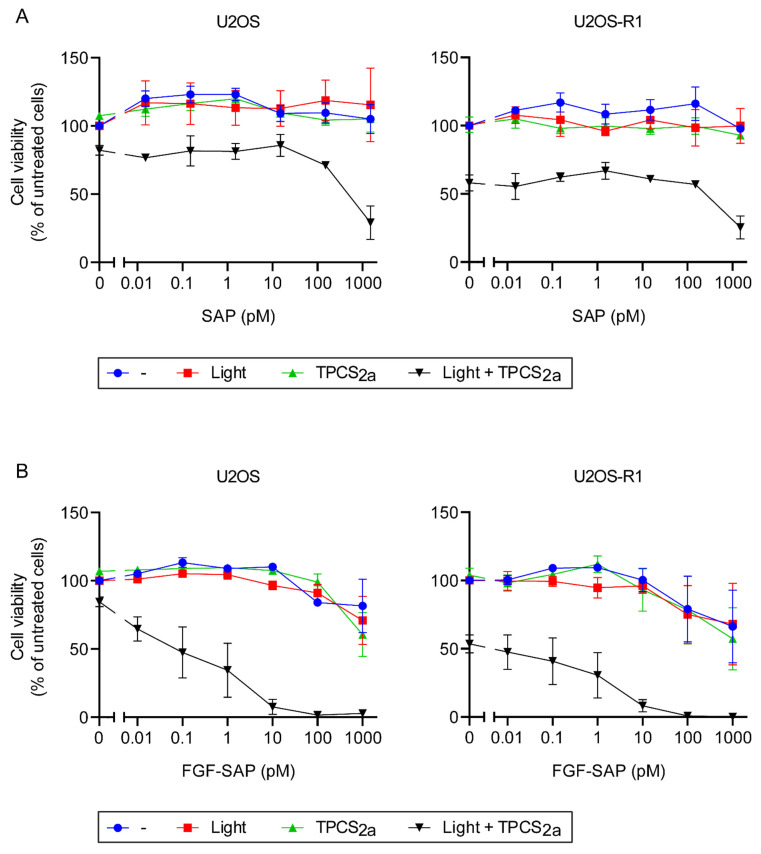
Cytotoxic effects of SAP (**A**) and FGF-SAP (**B**) with or without PCI in U2OS and U2OS-R1 cells. Cells were incubated with increasing concentrations of SAP or FGF-SAP for 18 h in the presence or absence of 0.2 µg/mL TPCS_2a_, washed and incubated further for four hours in drug- and TPCS_2a_-free medium. The cells were then exposed or not to blue light for 120 s and MTS were assessed 48 h later. The following conditions were tested: drug (SAP/FGF-SAP) alone (blue), drug and light exposure (red), drug and TPCS_2a_ (green), drug, TPCS_2a_ and light exposure (black). The data are presented as means relative to the mean of untreated cells (in the case of TPCS_2a_ treatment, cells without TPCS_2a_ were included for each sample and used for normalization). The graph represents the mean of three independent experiments with three replicate samples for each condition in each experiment. Error bars ± SEM, *n* = 3.

U2OS or U2OS-R1 cells were treated with increasing concentrations of SAP (Figure 3A) or FGF-SAP (Figure 3B), incubated for 18 h, washed and incubated further for 4 h in drug-free medium, and illuminated for 120 s in the presence 0.2 μg/mL TPCS_2a_ (photosensitizer). An additional 4 h incubation after washing was included for TPCS_2a_ to achieve endosomes and removal from the cell surface before illumination. As controls, cells were also treated with drug alone and no illumination (-), with drug and illumination (Light), or with drug and TPCS_2a_ but no illumination (TPCS_2a_). Unconjugated SAP served as a control for the targeted ligand toxin, FGF-SAP.

Treatment of cells with drug alone, either SAP (blue line in Figure 3A) or FGF-SAP (blue line in Figure 3B) did not induce cytotoxic effects in U2OS or U2OS-R1 cells, although a slight increase in cytotoxicity was observed for FGF-SAP at the highest concentrations of drug. Cells treated with drug, SAP or FGF-SAP, followed by illumination in the absence of photosensitizer (TPCS_2a_), was included in order to determine if light by itself affects the uptake or activity of SAP/FGF-SAP. In both cell lines, the effect of light, as an independent variable on SAP or FGF-SAP, had very little or no influence on cell viability (red line in Figure 3A,B). Moreover, incubation of cells, U2OS and U2OS-R1 with drug in the presence of photosensitizer (TPCS_2a_) but without illumination (green line Figure 3A,B), did not alter cytotoxicity considerably. However, in all cases, FGF-SAP alone demonstrated higher cytotoxicity than SAP alone toward both cell lines (compare Figure 3A,B). 

Despite the low susceptibility to SAP alone in both cell lines, PCI of cells treated with SAP reduced cell viability substantially (black line Figure 3A). Although PCI of SAP in U2OS-R1 cells was slightly more efficient than in U2OS cells, no considerable difference in cytotoxicity was observed between the two cell lines. The potential increase in cytotoxic efficiency of PCI of SAP in the case of U2OS-R1 could be an effect of the slightly higher uptake of photosensitizer in U2OS-R1 than in U2OS cells.

Additionally, PCI of cells treated with FGF-SAP strongly potentiated the cytotoxicity of the drug (black line Figure 3B) and was also considerably more cytotoxic than PCI of SAP. The viability was reduced to approximately 5% in cells treated with 10 pM FGF-SAP, while it was only reduced to 60–80% in the case of cells treated with the same molar ratio of SAP (as FGF-SAP preparations contain on average 1.5 molecules SAP per FGF2, 10 pM FGF-SAP equals 15 pM SAP). Clearly, PCI of FGF-SAP is more cytotoxic than PCI of SAP. Even though PCI greatly enhanced the effect of FGF-SAP, the difference in cytotoxic response between the cell lines was surprisingly low.

#### 3.3.2. Cytotoxic Effect of PCI after a Short (Four Hours) FGF-SAP Incubation

**Figure 4:**
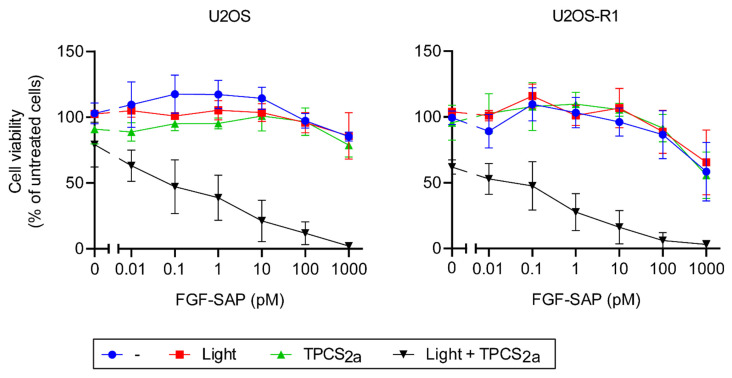
Cytotoxic effects of 4 h incubation of FGF-SAP with or without PCI in U2OS and U2OS-R1 cells. Cells were incubated with or without 0.2 μg/mL TPCS_2a_ for 18 h prior to 4 h incubation with photosensitizer-free medium containing increasing concentration of FGF-SAP. The cells were then exposed or not to blue light for 120 s and MTS was assessed 48 h later. The following conditions were tested: drug (SAP/FGF-SAP) alone (blue), drug and light exposure (red), drug and TPCS_2a_ (green), drug, TPCS_2a_ and light exposure (black). The data are presented as means relative to the mean of untreated cells (in the case of TPCS_2a_ treatment, cells without TPCS_2a_ were included for each sample and used for normalization). The graph represents the mean of three independent experiments with three replicate samples for each condition in each experiment. Error bars ± SEM, *n* = 3.

Since PCI of FGF-SAP was toxic toward both U2OS and U2OS-R1 when incubating cells with FGF-SAP for 18 h, we tested whether a shorter incubation time would have an effect on the cytotoxicity. We hypothesized that a shorter incubation with the drug might reveal potential differences in the cytotoxic efficiency of FGF-SAP in cells expressing FGF receptor (U2OS-R1) or not (U2OS). Cells were therefore incubated for 18 h with or without 0.2 μg/mL TPCS_2a_, washed and incubated for additional four hours in the presence of increasing concentrations of FGF-SAP, ranging from 0.01 pM to 1000 pM (Figure 4).

Again, PCI of FGF-SAP increased the cytotoxic effect compared to FGF-SAP alone (Figure 4). PCI of cells treated with 10 pM FGF-SAP for 4 h reduced the viability to 15–20%. The cytotoxic response to a shorter incubation with FGF-SAP (4 h instead of 18 h) was not much altered. Moreover, a short incubation (four hours) of FGF-SAP in U2OS-R1 and U2OS cells combined with PCI also resulted in similar cell viability for both cell lines.

#### 3.3.3. Co-Incubation of FGF-SAP and Heparin

**Figure 5:**
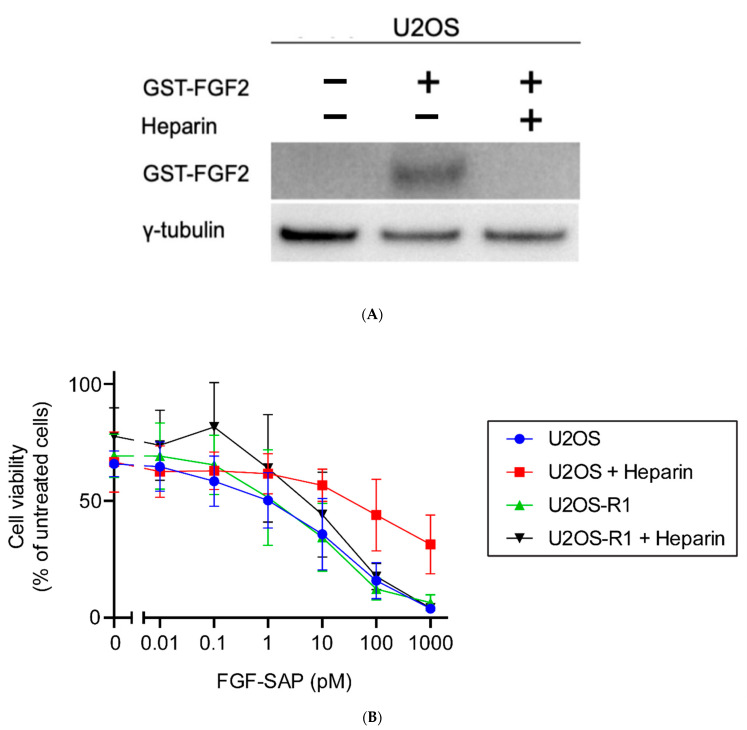
Involvement of HSPGs in binding and toxicity of FGF-SAP in U2OS. (**A**) Binding of GST-FGF2 in U2OS cells in the presence or absence of heparin. Cells were treated with 600 ng/mL GST-FGF2 in the presence or absence of 50 U/mL heparin, on ice for 1 h. Cells were then lysed, and the cellular material was analyzed by SDS-PAGE and Western blotting using the indicated antibodies. Representative image of one out of two experiments is shown. (**B**) Cytotoxic effects of PCI of FGF-SAP are reduced by co-treatment with heparin. Cells were incubated with 0.2 μg/mL TPCS2a for 18 h prior to 4 h incubation with photosensitizer-free medium containing increasing concentration of FGF-SAP with or without 20 U/mL heparin. The cells were then exposed for 120 s to blue light and MTS was assessed 48 h post light exposure. The data are presented as means relative to the mean of untreated cells. The graph represents the mean of three independent experiments with three replicate samples for each condition in each experiment. Error bars ± SEM, *n* = 3.

HSPGs play an important role in signaling through FGFRs and are abundant on the cell surface and in the extracellular matrix. We therefore hypothesized that HSPGs could mediate the internalization of FGF-SAP and, as such, account for the unspecific cytotoxic responses obtained in U2OS cells with very low levels of FGFR1. Heparin, a highly sulfated form of heparan sulfate, can outcompete binding of FGFs to HSPGs [40,41]. The role of HSPGs in association of FGF-SAP to U2OS cells was therefore investigated by treating U2OS cells with or without heparin and analyze binding of FGF2 to the cells by Western blotting. FGF2-Saporin was, however, not used for this purpose, due to its high toxicity. We instead used a surrogate, i.e., a fusion protein of FGF2 and glutathione-S-transferase (GST), GST-FGF2. Cells were treated with GST-FGF2 +/− heparin and incubated for 1 h on ice to allow binding, but to avoid endocytosis and degradation of GST-FGF2. Western blot analysis (Figure 5A) revealed that GST-FGF2 bound to U2OS cells in the absence, but not in the presence, of heparin.

We therefore tested the toxicity of FGF-SAP in the presence or absence of heparin in U2OS and U2OS-R1 cells. The cells were treated with increasing concentrations of FGF-SAP, ranging from 0.01 pM to 1000 pM, incubated for 4 h and illuminated for 120 s, in the presence of 0.2 μg/mL TPCS_2a_. In addition, in order to investigate HSPG-mediated internalization of FGF-SAP, 20 U/mL heparin was added to the culture medium, to reduce binding to cell-surface HSPGs.

The presence of heparin resulted in a reduced cytotoxic effect of PCI of FGF-SAP in U2OS cells, while heparin treatment had no effect on cytotoxicity of PCI of FGF-SAP in U2OS-R1 cells (Figure 5B). We conclude that heparin outcompetes binding of FGF-SAP to HSPGs at the cell surface of U2OS cells and thus prevent FGF-SAP from entering the cells. In U2OS-R1 cells, FGF-SAP is most likely taken up by binding to its high-affinity receptor, FGFR1, which is not inhibited by heparin. Actually, heparin is known to stabilize the complex of FGF2 and FGFR1 [10,12]. Therefore, there is little change in toxicity with or without heparin in this case (Figure 5B).

### 3.4. Association of FGF-SAP to U2OS Cells Assessed by Immunofluorescence

To investigate the role of HSPGs in the internalization of FGF-SAP, cells were examined by confocal microscopy. Due to the toxicity issues of FGF2-SAP, we used GST-FGF2 in these experiments. Cells were treated with GST-FGF2, with or without heparin, for 4 h. Chloroquine (CQ) was added to prevent lysosomal degradation. Cells were then fixed and stained with a marker for lysosomes (LAMP1) in addition to a nuclear dye (Hoechst).

**Figure 6:**
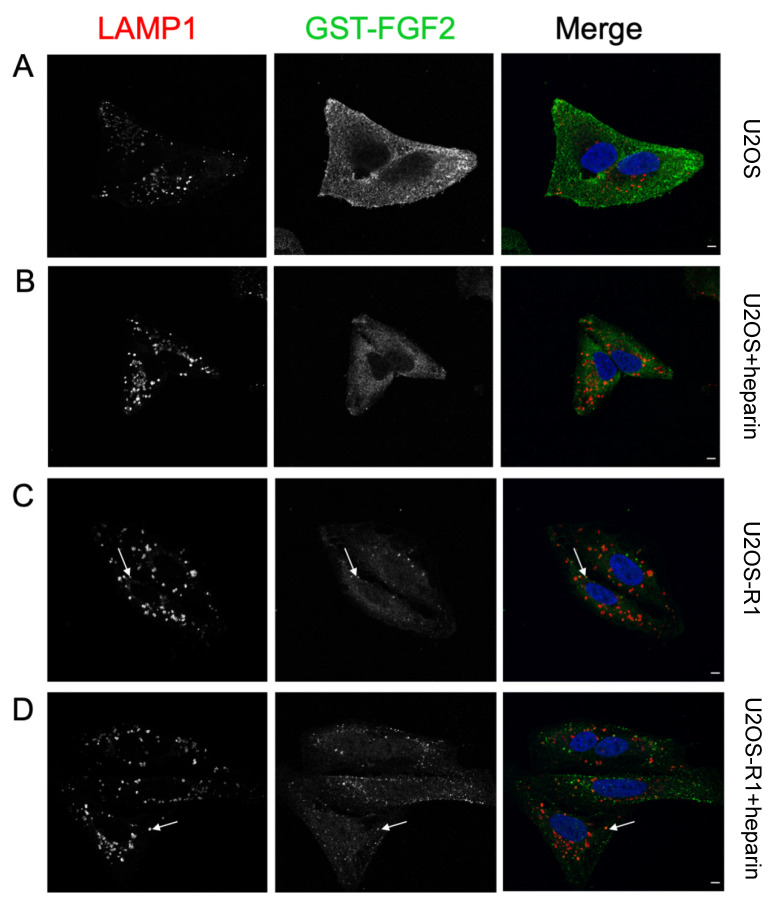
Localization of LAMP1 and GST-FGF2 in U2OS and U2OS-R1 cells. (**A**–**D**) Cells were incubated with CQ for 1 h, prior to a 4 h incubation with 100 ng/mL GST-FGF2 +/− 20 U/mL heparin at 37 °C, 5% CO_2_. Cells were then fixed and stained for LAMP1 (red), GST-FGF2 (green) and nucleus (blue). (**A**) U2OS − Heparin (**B**) U2OS + Heparin (**C**) U2OS-R1 -Heparin (**D**) U2OS-R1 + Heparin. Arrows point to examples of colocalization. Scale bar, 5 µm. Images from one representative experiment (out of three) are presented.

There was a considerable reduction in the fluorescent signal representing GST-FGF2 (green) in U2OS cells treated with GST-FGF2 and heparin (Figure 6B) in contrast to U2OS cells treated with GST-FGF2 alone (Figure 6A), indicating that heparin efficiently competes out the binding to cell-surface HSPGs. In the case of U2OS-R1 cells, GST-FGF2 was internalized well in the presence and absence of heparin (Figure 6C,D), probably through FGFR1. The distribution of GST-FGF2 in U2OS and U2OS-R1 cells was different. U2OS-R1 cells displayed GST-FGF2in typical endosomal structures while GST-FGF2 was more dispersed in U2OS cells. Partial colocalization of GST-FGF2 and LAMP1 was observed in U2OS-R1 cells (Figure 6C,D), but not in U2OS cells (Figure 6A,B). We also observed slightly enlarged lysosomal structures that are probably a result of CQ treatment. Taken together, the data indicate that FGF-SAP binds and may be internalized by HSPGs in U2OS cells.

## 4. Discussion

In the present study, we evaluated PCI of FGF-SAP as a strategy to target and achieve cytotoxic effects aimed specifically at cancer cells overexpressing FGFRs. After establishing optimal photochemical doses for PCI in our cellular system, we demonstrated that PCI of FGF-SAP induced strong cytotoxic effect. However, FGF-SAP was not specific to FGFRs. Our data suggests that the unspecificity of FGF-SAP could be due to HSPG-mediated binding and internalization of FGF-SAP in U2OS cells. The results suggest that FGF-SAP was internalized by HSPGs by showing that heparin (1) decreased the cytotoxicity of PCI of FGF-SAP, (2) decreased the internalization of GST-FGF2 shown by immunofluorescence, and (3) blocked surface-binding of GST-FGF2 assessed by Western blotting. Taken together, this points to HSPG-mediated internalization of FGF-SAP as being the responsible mechanism for the highly cytotoxic effects observed in U2OS cells. The results further suggest, due to strong PCI-induced cytotoxicity, that the present type of FGF-SAP is not specific enough to be combined with PCI unless binding to HSPGs can be omitted.

The role of HSPGs as coreceptors and regulators of signaling through FGFRs is well acknowledged [8,12]. Furthermore, membrane HSPGs have been reported as endocytic receptors that undergo constitutive as well as ligand-induced endocytosis [42,43]. The endocytic route of HSPGs is unclear [43]. The rate at which endocytosis occurs and the fate of the endocytic vesicle are dependent on cell type, the HS-binding ligand, and the roles and localization of the HSPG core protein on the cell membrane, as well as other key membrane-associated molecules [42,43]. Internalization of FGFs by HSPGs has been demonstrated both in vitro and in vivo [44,45,46,47]. Although there is a lack of research on this topic, HSPG-mediated internalization of FGF2 has been shown to proceed at a slower rate compared to that of FGFR1 [48,49]. This could explain why there is a lack of colocalization of GST-FGF2 with LAMP1 in U2OS cells when observed at the same timepoint in U2OS-R1 cells.

The findings presented here constitute a major challenge, as HSPGs exist on most, if not all, cell types. Therefore, in order to specifically target cancers overexpressing FGFRs using PCI, binding of SAP-FGF2 to HSPGs at the cell surface should be omitted. In this study, we attempted to do this by treating the cells with heparin. This is not therapeutically applicable, as it would require a high dose of heparin administrations and risk serious adverse events such as heparin-induced thrombocytopenia/thrombosis syndrome and bleeding [50]. A rational strategy to achieve improved selectivity towards FGFRs is to mutate the HS binding site of FGF2, thereby preventing binding to membrane HSPGs, in favor of FGFR-mediated internalization. 

In a clever approach, FGF-SAP has been used to clear cell populations for FGFR expressing cells. For instance, primary cultures of pancreatic islets were cleared for fibroblasts by using FGF-SAP [32]. Possibly, in these applications, co-incubating FGF-SAP with heparin could improve targeting and specificity.

Analysis of cell viability showed that the U2OS-R1 cells are slightly more sensitive to PDT than the U2OS parental cells. If this is due to an enhanced capacity of the FGFR-positive U2OS-R1 cells to take up TPCS_2a_, which was indicated by the flow cytometry data (20% increase of TPCS_2a_ in FGFR-positive compared to FGFR-negative U2OS cells) remains to be investigated. It is possible that cells overexpressing FGFR1 have higher endocytic activity and therefore take up more photosensitizer. On the other hand, the slightly increased uptake of TPCS_2a_ in U2OS-R1 cells compared to U2OS parental cells could also simply be a result of clonal variations, and more experiments are needed to elucidate this. In any case, these observations do not change the conclusions presented in this paper, as U2OS cells, which had somewhat less uptake of TPCS_2a_, was still highly sensitive to PCI-mediated FGF-SAP toxicity, presumably due to binding and internalization through HSPGs.

Although PCI was potentiating the effects of FGF-SAP, we discovered that FGF-SAP was not specific to cells expressing high levels of FGFRs, as FGF-SAP also bound to HSPG at the cell surface. Several previous reports concerning cytotoxicity of FGF-SAP demonstrated effects in cells expressing FGFRs, although these were not done in combination with PCI [33,51]. In these studies, the possible contribution of HSPG-mediated uptake of FGF-SAP was not evaluated. Our study indicates that control conditions, for instance cells with or without FGFRs, should be used.

The main conclusion of this study is that FGF-SAP, in its present form, should not be combined with any strategies that facilitate enhanced endosomal escape in a therapeutic setting, as high toxicity unspecific to FGFRs could be the result.
